# Seasonal Survival Probabilities Suggest Low Migration Mortality in Migrating Bats

**DOI:** 10.1371/journal.pone.0085628

**Published:** 2014-01-15

**Authors:** Simone Giavi, Marco Moretti, Fabio Bontadina, Nicola Zambelli, Michael Schaub

**Affiliations:** 1 Community Ecology, Swiss Federal Research Institute WSL, Bellinzona, Ticino, Switzerland; 2 Urban Ecology and Wildlife Research, SWILD, Zurich, Switzerland; 3 Biodiversity and Conservation Biology, Swiss Federal Research Institute WSL, Birmensdorf, Zurich, Switzerland; 4 Museo Cantonale di Storia Naturale, Lugano, Ticino, Switzerland; 5 Swiss Ornithological Institute, Sempach, Luzern, Switzerland; Università degli Studi di Napoli Federico II, Italy

## Abstract

Migration is adaptive if survival benefits are larger than costs of residency. Many aspects of bat migration ecology such as migratory costs, stopover site use and fidelity are largely unknown. Since many migrating bats are endangered, such information is urgently needed to promote conservation. We selected the migrating Leisler's bat (*Nyctalus leisleri*) as model species and collected capture-recapture data in southern Switzerland year round during 6 years. We estimated seasonal survival and site fidelity with Cormack-Jolly-Seber models that accounted for the presence of transients fitted with Bayesian methods and assessed differences between sexes and seasons. Activity peaked in autumn and spring, whereas very few individuals were caught during summer. We hypothesize that the study site is a migratory stopover site used during fall and spring migration for most individuals, but there is also evidence for wintering. Additionally, we found strong clues for mating during fall. Summer survival that included two major migratory journeys was identical to winter survival in males and slightly higher in females, suggesting that the migratory journeys did not bear significant costs in terms of survival. Transience probability was in both seasons higher in males than in females. Our results suggest that, similarly to birds, Leisler's bat also use stopover sites during migration with high site fidelity. In contrast to most birds, the stopover site was also used for mating and migratory costs in terms of survival seemed to be low. Transients' analyses highlighted strong individual variation in site use which makes particularly challenging the study and modelling of their populations as well as their conservation.

## Introduction

Migration is a response to seasonal fluctuations of resources [1]. While reproduction occurs at places and at times where and when resources for rearing young are maximal, these places may not be ideal for survival at other times of the year. Therefore animals migrate to locations where survival is maximised during part of the year. Although migration can be beneficial compared to residency it bears costs in terms of energy consumption and ultimately of survival. Thus, animals have to trade-off costs and benefits of migratory behaviour in order to achieve maximal fitness. Migratory costs in terms of survival can be substantial. Among birds, the black-throated blue warbler *Dendroica caerulescens* has a mortality rate 15 times higher during the migratory period compared to the stationary period [2]. Nonetheless, there is generally little empirical-based knowledge about migratory cost and more specifically about mortality related to migration [3].

Migratory behaviour is known to occur in bats, but our knowledge about the migration ecology of bats is limited compared to that of other mammals and birds in particular [3]. Some bat species perform large-distance migrations between summer roosts and hibernacula [4]. Migratory movements of up to 1500 km have been recorded based on marked individuals in some European bat species [5], [6]. There is also evidence that females of some species conduct longer journeys than males, suggesting that males stay at locations near wintering places or on the migration route, without returning to the nursery colonies [7]. Several studies showed that migrating bats could use one or several sites on migration routes to stop over for resting and foraging before continuing the journey [8], [9], [10]. Because bats can save energy by daily torpor, it seems that they need less energy than similar sized birds during the time spent at stopover sites [10].

Survival in bats is little known so far, and the main focus of past studies has been to estimate annual survival probabilities and to model the factors possibly involved [11], [12]. As far as we know, there are only two studies that investigated seasonal survival with the goal to assess whether different periods in the life cycle are differentially dangerous for bats. Sendor and Simon [13] focussed on a population of Pipistrelle bat (*Pipistrellus pipistrellus*) that cover only short distance movements between breeding and hibernating sites. They found little seasonal variability of the estimated survival. Papadatou et al. [14] found similar estimates of survival during summer and winter in long-fingered bats (*Myotis capaccinii*), but also this species conduct migration journey over short distances only. Since studies on seasonal survival in long-distance migrating bats are lacking, it is unknown whether migratory periods are associated with increased mortality compared to stationary ones.

In our study we investigated the seasonal survival of Leisler's bat *Nyctalus leisleri*, a migratory forest dwelling bat, to understand whether migratory journeys were associated with increased mortality compared to stationary periods and to identify site fidelity at the study area. Ring recoveries and phenological studies on Leisler's bat suggest that populations reproducing in Central and Northern Europe migrate to places south of the Alps or South-Western Europe [15], [16], [17], [18]. Most males do not return to the breeding areas but stay either in the wintering areas or at sites between the two. Apparent survival of juvenile males at a nursery site in southern Thuringia was very low indicating strong natal dispersal [11].

We collected capture-recapture data at a non-reproducing site in southern Switzerland year round from 2001 to 2006 and estimated seasonal survival during winter and during summer. Females that appear at out study site likely reproduce in Germany about 1000 km further north [5]. A fraction of them spend the winter at our study site, but some also migrate further south for wintering. Thus, summer survival includes two long migratory journeys with the crossing of the Alps, while winter survival includes, if anything, only short migratory journeys. The migration of males is less well understood. Most of them likely spend the summer somewhere between our study site and the reproducing sites of females, thus conduct shorter migratory journeys than females [6], [11], [21]. If migrations bear significant costs in terms of survival, we expect survival to be lower during summer than during winter (in particular in females) and differences in seasonal survival to be stronger in females compared to males. Subsequently we quantified the site fidelity of Leisler's bats to the study area. This was achieved by additionally estimating the transience probability of the captured individuals at the study site, i.e. the probability that an individual never comes back to the study area after marking, and by comparing annual survival estimates in our study area with those estimated in a reproduction area in southern Thuringia (Germany) [11]. If transience probability is low and the annual survival estimates in our study are similar to the annual survival estimates from reproduction area, we conclude that bats are faithful to the study area across several years (i.e. high site fidelity). Finally, we assessed the functional role of the study site in the migration of Leisler's bats. We were interested in understanding whether the area was used for mating, and whether it served exclusively as a wintering site used from autumn to the next spring or whether it is a stopover site used in autumn and/or in spring for a limited period of time only.

## Materials and Methods

### Study species

The Leisler's bat is a temperate bat species with body mass ranging from 13 to 20 g. In Central Europe it is a tree-dwelling bat which forages mainly above the canopy of different types of forests [19], [20]. Like other *Nyctalus* species, Leisler's bats show a sexually differential migratory behaviour [6]. During the reproducing season in summer, females aggregate in nursery colonies that are located in tree cavities or artificial bat boxes which are often clustered within a limited area [21]. Nursery colonies are mainly composed by females with their juveniles from April to beginning of October. These individuals then migrate to wintering areas in South-Western Europe covering distances of about 1000–1500 km [5], [17]. Stopover sites are supposed to be interspersed along the migratory routes. The mating system of the Leisler's bat *Nyctalus leisleri* is described as resource defence polygyny [22]. Males occupy roosts in open woodlands and attract up to ten females by songflights, thus the roosts have the function of lekking arenas [23], [24]. Female Noctule bats may switch among several roosts within mating seasons and there is evidence that they mate with several males [23].

### Data sampling

We conducted the study in Alto Malcantone, Canton of Ticino, Switzerland (46°03′N, 8°53′E). The study area extends over 6 km^2^ in a mountainous region (altitudinal range 500–1′000 m a.s.l.) dominated by mixed deciduous forest (67%) mainly composed of European chestnut (*Castanea sativa*) with interspersed oak (*Quercus* sp.) and birch (*Betula* sp.). Mean temperature in January is 3.4°C and in July 22.2°C (data from 1985 to 2012, [25]).

We surveyed Leisler's bats in 200 bat boxes (all produced by Schwegler, Germany; www.schwegler-natur.de; 150 boxes were type 2F, 50 boxes type FN) that were set in the study area between 1999 and 2006. Bat boxes were opened and checked for presence-absence of bats from April 2001 to December 2006. Most controls were performed during autumn and spring (Table S1 in File S1). During 54 controls, all bats in the boxes were checked, recorded, and tagged with forearm bands. We recorded sex and reproductive status (spermatogenesis for males and lactation for females) according to Racey [26], but none of the captured individuals were lactating indicating that the study area was not used for reproduction. We also intended to determine the age according to Anthony [27], but we only detected individuals with fully calcificated phalanges, implying that individuals born in the same year either develop their phalanges before the start of the migration or that they do not appear in our study area. We think that the former explanation is much more likely than the latter. In any case we did not consider an age structure in the analyses.

Bat boxes installation, sampling and marking have been conducted with the authorization of the Federal Veterinary Office, the Federal Environmental Office and the Forest Department of the Canton of Ticino. All measures to minimize disturbance and ameliorate suffering to the animals have been taken.

### Data analysis

To estimate seasonal survival we considered captures exclusively collected in non-reproductive and non-hibernating periods, i.e. from 16-Aug to 15-Nov (autumn captures) and from 16-Feb to 15-May (spring captures) of each year and pooled all captures obtained within these resulting in 12 capture occasions. We have selected these two seasonal capture periods because they corresponded to the main activity periods of Leisler' bats at the site [17] and because we conducted most bat box controls in these periods (Table S1 in File S1). We constructed individual capture histories, which is a matrix whose columns correspond to capture occasions whereas the rows correspond to marked individuals. Matrix entries are binary with 1 indicating the capture of a specific individual at a given capture occasion and 0 otherwise.

The data were analysed with the Cormack-Jolly-Seber (CJS) model which separately estimates the probabilities of apparent survival (*φ*) and of recapture (*p*) [28]. The recapture probability is the probability that a marked individual that is alive and present in the study area at sampling occasion *t* is captured at sampling occasion *t*. The apparent survival probability is the probability that a marked individual alive at sampling occasion *t* survived and has not permanently emigrated between sampling occasions *t* and *t+*1. Mortality and permanent emigration are confounded and consequently the estimate is lower than true survival. Since we considered autumn and spring capture occasions, we were able to estimate survival from spring to autumn (summer survival), which includes the reproduction period and, at least for females, the two major migratory journeys between nursery colonies and the study area and from autumn to spring (winter survival) which includes the period of hibernation and potential migratory journeys from the study area to wintering areas. Each of these seasonal survival estimates refers to a period of six months. The capture histories were summarized in the *m*-array format and were analysed with the multinomial distribution, whose parameters are functions of survival and recapture probabilities [28], [29].

In order to infer whether or not the study area was used as a mating site we looked for the occurrence of harems within bat boxes. During mating males do not tolerate each other in the same box [11], thus a harem is the grouping of one male and two or more females in a bat box. We computed the proportion of males in bat boxes and inferred from a low proportion the occurrence of harems. We used the number of males and females in each bat box that was occupied by at least one male and one female during each control and built a binomial model to estimate the proportion of males in the boxes in each season. Additionally we computed the probability that the proportion of males in a given season is larger than in the other seasons.

Finally we assessed seasonal phenology using the number of males and females captured during each control. We built a Poisson model to estimate for each sex the mean number of captured individuals per control during each season.

### Basic structure of the CJS model

We used a variant of a CJS model that included a parameter for transience and that considered random temporal effects for survival and transience. The most complex model considered fixed effects of sex and time for recapture probabilities (*p*). Since there were two seasonal capture periods in each of the six years, an effect of time can equally be expressed as an interacting effect of season (autumn, spring) and year. This formulation also allows the possibility to model additive effects, pure seasonal or pure year effects. For survival we included random time effects, that is the time-specific survival probabilities were considered to be the realisation of a stochastic process with sex-specific mean 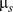 and temporal variance 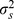:
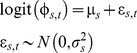
where *s* is an index for sex and *t* for time. We used a similar stochastic process to integrate transience (*τ*) into the model:
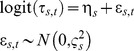
where 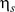 and 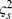 are the sex-specific mean and variance of transience probability, respectively, and *s* and *t* indices for sex and time. We estimated transience probability *τ* by fitting an age structure to the survival model [30]. The age structure had two age classes, where survival of the first age class (*φ*_1_) refers to survival just after first capture, while survival of the second age class (*φ*_2_) refers to all later survival periods. The probability that a newly caught individual is a transient is then *τ* = 1−*φ*_1_/*φ*_2_. Usually the two survival probabilities are modelled directly, while *τ* is a derived parameter. However, we reparameterised the model in such a way that our focal parameters, *τ* and *φ*_2_, were directly modelled and *φ*_1_ became a derived parameter. Hereafter we term *φ*_2_ = *φ*, since this is the parameter of our interest (apparent survival of non-transients).

The Cormack-Jolly-Seber model requires several assumptions to be satisfied [28], which we tested with a goodness-of-fit test. The goodness-of-fit test performed with U-CARE [31] of the transient model *φ*(sex*season*year), *τ*(sex*season*year), *p*(sex*season*year) did not indicate lack of fit (*χ* ^2^ = 63.02, df = 51, *P* = 0.12).

### Candidate models and model selection

In order to reduce the number of models to be analyzed while accounting for possible factors combinations, we proceeded with a three steps approach:

Step 1 - We first modelled recapture probabilities (*p*) by considering all possible effects of sex, season and year. The survival and the transient models were kept at their most complex structure. Step 2 - We then evaluated whether sex was important for survival and transience by fitting models with and without sex effects for these parameters. We always used the recapture model that fitted the data best as identified in step 1, i.e. which was the most parsimonious for recapture. Step 3 – We quantified the seasonal effects on survival and transience. To do that, we decomposed the temporal variation into a fixed seasonal and a random annual component. We included this decomposition either only in one sex, in both sexes or in none in order to evaluated potential sex-specific differences. We calculated annual survival probability from seasonal survival probabilities by their product.

The models were analysed using Bayesian methods with JAGS [32]. JAGS was called from R [33] using package R2jags [34]. Two different Markov chains, starting at random initial values in the range of parameter space, were run during 200′000 iterations and the initial convergence phase was excluded by dropping the first 25′000 iterations. We thinned Markov chains with a factor of hundred and used the Brooks-Gelman-Rubin criterion 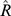 [35] to assess the convergence of chains to a stationary distribution. We specified flat prior distributions for target variables, namely uniform distributions on [0, 1] for mean survival, transience and detection probabilities and uniform distributions on [0, 5] for the standard deviations. The code of the most complex model is available in Text S1.

We ranked the different models (Table S2 in File S1) using the deviance information criterion DIC [36]. The DIC is considered as the Bayesian counterpart to the Akaike information criterion (AIC) that is used for model selection in the maximum likelihood approach [37]. For making inference we averaged the parameters of models from the third model selection step according to [38]. The resulting average is indicated with 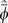 and is the sum of the products of each single estimate and the corresponding model weight. More formally
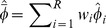
where 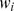 are the model weights (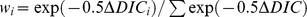) and 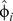 the posterior means of parameter 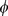 of each of the *R* candidate models.

## Results

**Figure 1:**
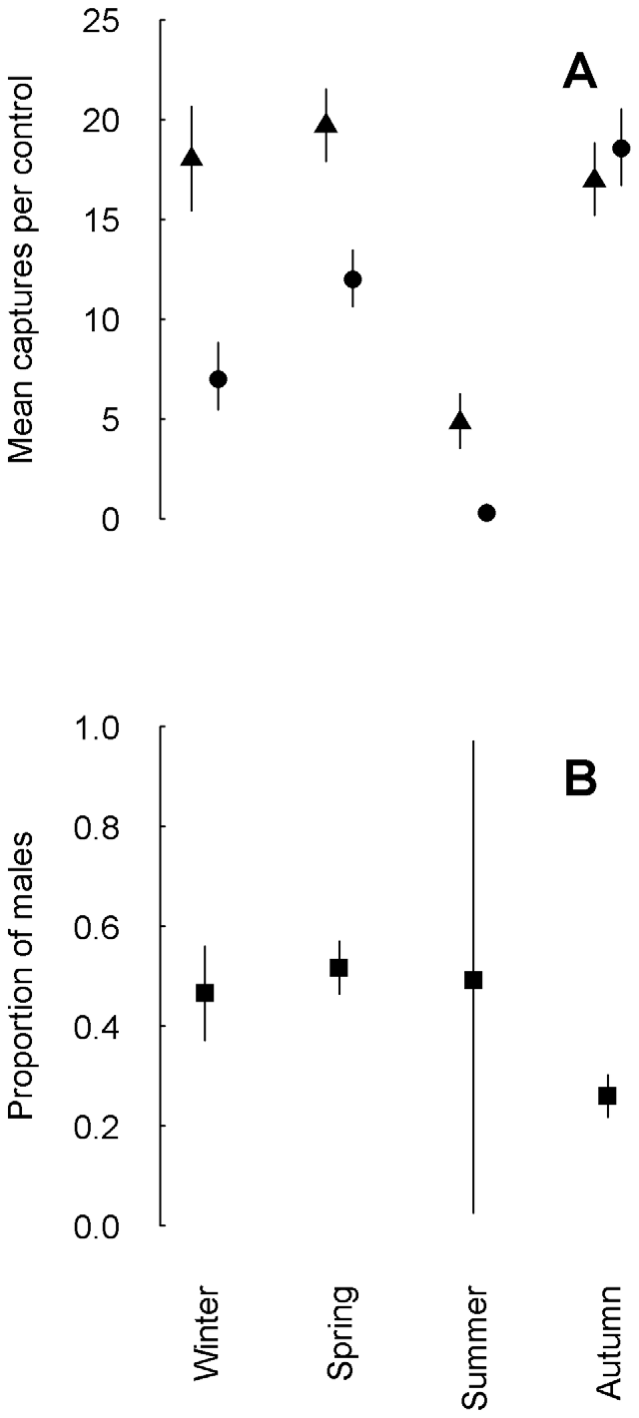
Mean number of captures per control and proportion of males within boxes. Mean number of captured Leisler's bat (*Nyctalus leisleri*) individuals per complete control (A) (triangles males, dots females) and mean proportion of males in boxes (B) in four seasons from 2001–2006 in southern Switzerland. Winter refers to the period from November 16th to February 15th, spring to the period from February 16th to May 15th, summer to the period from May 16th to August 15th and autumn to the period from August 16th to November 15th. The vertical lines show the limits of the 95% credible intervals.

From our 6 years capture-recapture study, we obtained a total of 1643 captures of Leisler's bats from a total of 461 marked individuals. The yearly phenology indicated that box occupancy differed between sexes (Fig. 1, panel A). A similar number of males was present during autumn, winter and spring, while during summer the number was much lower. Female numbers raised in spring and peaked in autumn. During winter fewer females were present and during summer they were almost absent. Since neither juveniles nor lactating females were present in summer, the study site was not used for breeding. The mean proportion of males within controlled boxes was around 0.5 in all seasons but in autumn (Fig. 1, panel B). The probability that the proportion of males in the boxes was lower in autumn than in the other seasons was 1.

### Modelling recapture probability (p)

For the survival analyses we used 389 marked individuals (164 males, 225 females), as they were captured in the defined autumn and spring capture occasions. Of these 92 males (56%) and 74 females (33%) were recaptured at least once in a subsequent capture occasion.

The ranking of the different models for the recapture probability was unambiguous (Table 1). The most complex model that included a different recapture probability for each sex and capture occasion was the best in terms of the DIC, suggesting that recapture probability was very heterogeneous ranging between 0.083 and 0.857 (Fig. S1). This structure of *p* has been used for modelling the structure of *φ* and *τ*.

**Table 1:** Model selection for recapture probabilities of Leisler's bat (*Nyctalus leisleri*) in southern Switzerland during the period 2001–2006.

Recapture model	Deviance	pD	ΔDIC	w_i_
*p*(sex * year * season)	378.96	31.58	0.00	0.54
*p*(year * season)	385.50	26.69	1.64	0.24
*p*(year + season)	389.20	24.15	2.81	0.13
*p*(sex + [year * season])	386.64	28.73	4.82	0.05
*p*(sex + year + season)	390.20	25.50	5.15	0.04
*p*(sex * year)	398.28	24.06	11.80	0.00
*p*(year)	404.27	21.28	15.01	0.00
*p*(sex + year)	405.57	23.49	18.51	0.00
*p*(season)	413.56	19.94	22.95	0.00
*p*(sex + season)	414.20	20.31	23.96	0.00
*p*(sex * season)	414.06	21.10	24.62	0.00
*p*(sex)	421.37	17.56	28.37	0.00
*p*(.)	421.15	17.82	28.41	0.00

Given are the model names (Recapture model), the model deviance (Deviance), the model complexity (pD), the difference of the deviance information criterion between the current and the best model (ΔDIC) and the model weight (w_i_). The models are ordered from the best (lowest ΔDIC) to the worst one.

### Modelling survival (*φ*) and transience probabilities (τ)

We first evaluated whether survival and transience differed between sexes and whether the temporal variance was the same in both sexes. Model selection revealed that the two best models received similar support from the data (ΔDIC<0.13; Table 2). A common feature in these top ranked models was that both, survival and transience, included an effect of sex. There was uncertainty about whether the temporal variation of both parameters (survival and transience) differed between sexes.

**Table 2:** Model selection results for survival (Survival model) and transience (Transience model) probabilities of Leisler's bat (*Nyctalus leisleri*) in southern Switzerland, during the period 2001–2006.

Survival model	Transience model	Deviance	pD	ΔDIC	w_i_
μ_s_ + ε_s,t_; ε_s,t_ ∼ N(0,σ^2^_s_)	η_s_ + ω_s,t_; ω_s,t_ ∼ N(0,ς^2^_s_)	378.73	31.74	0.00	0.24
μ_s_ + ε_s,t_; ε_s,t_ ∼ N(0,σ^2^_s_)	η_s_ + ω_s,t_; ω_s,t_ ∼ N(0,ς^2^)	380.37	30.22	0.13	0.22
μ_s_ + ε_s,t_; ε_s,t_ ∼ N(0,σ^2^)	η_s_ + ω_s,t_; ω_s,t_ ∼ N(0,ς^2^_s_)	379.28	32.23	1.05	0.14
μ_s_ + ε_s,t_; ε_s,t_ ∼ N(0,σ^2^)	η_s_ + ω_s,t_; ω_s,t_ ∼ N(0,ς^2^)	380.88	31.63	2.05	0.09
μ_s_ + ε_s,t_; ε_s,t_ ∼ N(0,σ^2^)	η + ω_s,t_; ω_s,t_ ∼ N(0,ς^2^)	383.31	29.37	2.22	0.08
μ_s_ + ε_s,t_; ε_s,t_ ∼ N(0,σ^2^_s_)	η + ω_s,t_; ω_s,t_ ∼ N(0,ς^2^)	382.91	29.80	2.25	0.08
μ + ε_s,t_; ε_s,t_ ∼ N(0,σ^2^_s_)	η + ω_s,t_; ω_s,t_ ∼ N(0,ς^2^)	382.98	30.10	2.62	0.06
μ + ε_s,t_; ε_s,t_ ∼ N(0,σ^2^_s_)	η_s_ + ω_s,t_; ω_s,t_ ∼ N(0,ς^2^)	381.57	33.73	4.83	0.02
μ + ε_s,t_; ε_s,t_ ∼ N(0,σ^2^)	η + ω_s,t_; ω_s,t_ ∼ N(0,ς^2^)	384.54	31.00	5.09	0.02
μ_s_ + ε_s,t_; ε_s,t_ ∼ N(0,σ^2^)	η + ω_s,t_; ω_s,t_ ∼ N(0,ς^2^_s_)	382.11	34.16	5.81	0.01
μ + ε_s,t_; ε_s,t_ ∼ N(0,σ^2^_s_)	η_s_ + ω_s,t_; ω_s,t_ ∼ N(0,ς^2^_s_)	380.85	36.10	6.49	0.01
μ_s_ + ε_s,t_; ε_s,t_ ∼ N(0,σ^2^_s_)	η + ω_s,t_; ω_s,t_ ∼ N(0,ς^2^_s_)	382.02	34.95	6.51	0.01
μ + ε_s,t_; ε_s,t_ ∼ N(0,σ^2^_s_)	η + ω_s,t_; ω_s,t_ ∼ N(0,ς^2^_s_)	382.78	34.86	7.17	0.01
μ + ε_s,t_; ε_s,t_ ∼ N(0,σ^2^)	η_s_ + ω_s,t_; ω_s,t_ ∼ N(0,ς^2^)	384.16	33.77	7.47	0.01
μ + ε_s,t_; ε_s,t_ ∼ N(0,σ^2^)	η + ω_s,t_; ω_s,t_ ∼ N(0,ς^2^_s_)	384.96	34.71	9.20	0.00
μ + ε_s,t_; ε_s,t_ ∼ N(0,σ^2^)	η_s_ + ω_s,t_; ω_s,t_ ∼ N(0,ς^2^_s_)	384.05	38.25	11.84	0.00

Given the model names, the model deviance (Deviance), the model complexity (pD), the difference of the deviance information criterion between the current and the best model (ΔDIC) and the model weights (w_i_). The recapture model was in always the same, i.e. *p*(sex * year * season). For each survival and transient model the linear equation on the logit scale is given. μ_s_: mean survival for each sex; σ^2^_s_: temporal variance of survival for each sex; η_s_: mean transience for each sex; ς^2^_s_: temporal variance of transience for each sex.

**Figure 2:**
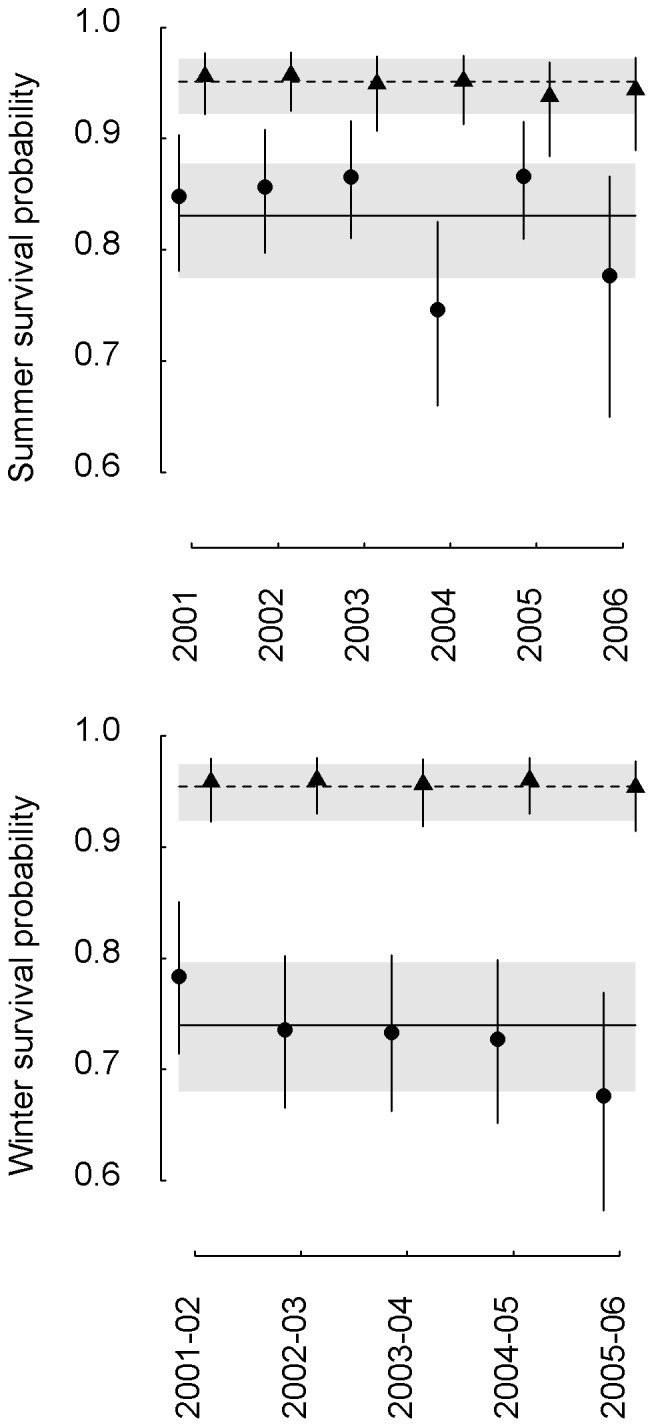
Summer and winter survival probabilities. Model averaged survival probabilities of male (triangles) and female (dots) Leisler's bats (*Nyctalus leisleri*) in summer (6 months from March to September) and winter (6 months from Septermber to March). The vertical lines show the limits of the 95% credible intervals. The horizontal lines show the mean seasonal estimates with the 95% credible intervals (shaded areas).

Model selection results of the decomposition of the temporal variance showed that several models received similar support by the data. There was support for a consistent seasonal difference in survival for females, while there was none for such a difference for males (Table 3). Moreover, a seasonal difference in transience was more justified by the data for females than for males. Model-averaged survival probabilities of males were nearly identical in both seasons (winter: 0.954; 95% credible interval, CI: 0.923–0.974; summer: 0.951; CI: 0.921–0.972; Fig. 2), and the annual survival probability was high (0.910; CI: 0.859–0.946). By contrast, model-averaged survival probabilities in females were lower during winter (0.740; CI: 0.679–0.796) than during summer (0.831; CI: 0.774–0.879; Fig. 2). The annual survival probability of females (0.613; CI: 0.544–0.680) was much lower than in males. Model-averaged transience probabilities of males were similar for individuals first captured in spring (0.464; CI: 0.395–0.522) and in autumn (0.480; CI: 0.409–0.539). In females, transience probabilities were lower in spring (0.191; CI: 0.126–0.269) than in autumn (0.296; CI: 0.207–0.381), and they were generally lower than in males.

**Table 3:** Model selection results for survival (Survival model) and transience (Transience model) probabilities of Leisler's bat (*Nyctalus leisleri*) sampled in southern Switzerland, during the period 2001–2006 in relation to seasonality.

Survival model	Transience model	Deviance	pD	ΔDIC	w_i_
μ_s_ + ε_s,t_; ε_s,t_ ∼ N(0,σ^2^_s_)	η_s_ + λ_f_ + ω_s,t_; ω_s,t_ ∼ N(0,ς^2^)	378.83	28.87	0.00	0.20
μ_s_ + γ_f_ + ε_s,t_; ε_s,t_ ∼ N(0,σ^2^_s_)	η_s_ + λ_f_ + ω_s,t_; ω_s,t_ ∼ N(0,ς^2^)	378.98	29.45	0.73	0.14
μ_s_ + γ_f_ + ε_s,t_; ε_s,t_ ∼ N(0,σ^2^_s_)	η_s_ + ω_s,t_; ω_s,t_ ∼ N(0,ς^2^)	379.00	30.28	1.58	0.09
μ_s_ + ε_s,t_; ε_s,t_ ∼ N(0,σ^2^_s_)	η_s_ + λ_f_ + ω_s,t_; ω_s,t_ ∼ N(0,ς^2^_s_)	377.99	31.43	1.72	0.08
μ_s_ + γ_f_ + ε_s,t_; ε_s,t_ ∼ N(0,σ^2^_s_)	η_s_ + λ_f_ + ω_s,t_; ω_s,t_ ∼ N(0,ς^2^_s_)	377.15	32.90	2.34	0.06
μ_s_ + γ_f_ + ε_s,t_; ε_s,t_ ∼ N(0,σ^2^_s_)	η_s_ + λ_m_ + ω_s,t_; ω_s,t_ ∼ N(0,ς^2^)	379.57	30.55	2.42	0.06
μ_s_ + ε_s,t_; ε_s,t_ ∼ N(0,σ^2^_s_)	η_s_ + ω_s,t_; ω_s,t_ ∼ N(0,ς^2^)	380.12	30.47	2.89	0.05
μ_s_ + γ_s_ + ε_s,t_; ε_s,t_ ∼ N(0,σ^2^_s_)	η_s_ + λ_f_ + ω_s,t_; ω_s,t_ ∼ N(0,ς^2^)	379.99	31.29	3.58	0.03
μ_s_ + γ_f_ + ε_s,t_; ε_s,t_ ∼ N(0,σ^2^_s_)	η_s_ + λ_s_ + ω_s,t_; ω_s,t_ ∼ N(0,ς^2^_s_)	377.71	33.72	3.73	0.03
μ_s_ + ε_s,t_; ε_s,t_ ∼ N(0,σ^2^_s_)	η_s_ + ω_s,t_; ω_s,t_ ∼ N(0,ς^2^_s_)	379.00	32.57	3.87	0.03

Only the best models are shown, a complete list of models is shown in Table S2 in File S1). Given are the model names, the model deviance (deviance), the model complexity (pD), the difference of the deviance information criterion between the current and the best model (ΔDIC) and the model weights (w_i_). The recapture model was always the same, i.e. *p*(sex * year * season). For each survival and transient model the linear equation on the logit scale is given. μ_s_: mean survival for each sex; γ_s_: fixed seasonal effect on survival for each sex; σ^2^_s_: temporal variance of survival for each sex; η_s_: mean transience for each sex; λ_s_: fixed seasonal effect on transience for each sex; ς^2^_s_: temporal variance of transience for each sex; m: parameter refers to males only; f: parameter refers to females only.

## Discussion

Our study aimed to improve the understanding of the migratory behaviour and site fidelity of Leisler's bats by investigating seasonal aspects in survival and using advanced Bayesian analytical techniques. We found distinct differences in seasonal survival between sexes, with males having generally high survival probabilities without seasonal differences, while females had lower survival probabilities with a seasonal pattern. Females survived better during the summer period which included two major migratory journeys than during the winter period, suggesting that migrations in Leisler's bat were not associated with significantly increased mortality. Differences in transience probabilities between sexes indicate differential fidelity to the studied site. We also provide evidence that the study site on the southern slope of the Alps was used during the non-breeding period likely acting as stopover site for most individuals. The majority of females showed high site fidelity and used the site during several years, while about only half of the males used it consistently. Thus, there was individual and sex-based heterogeneity in the use of this study area.

### Evidence for low migratory costs in terms of survival

There are few studies quantifying potential costs of migration in terms of survival in birds [2], [39], [40], [41], but, as far as we know, no study has ever tried to estimate migration costs in bats. We expected to observe lower survival in summer than in winter and generally lower survival in obligate migrating females than in males if migration is costly. Summer survival of both sexes was not lower than winter survival, which contradicts the migration cost we hypothesized. However, summer survival of females was indeed lower than that of males, which is in favour of the migration cost hypothesis.

Based on our results, we can quantify the maximal possible mortality that occurred during the migration, if we assume that survival probability during the non-migratory period in summer is one (i.e. no mortality during the stationary period in the breeding area). In this case the highest mortality following autumn and spring migration would be about 0.17 in females and 0.05 in males, which is at maximum about 44% (1−0.83)/(1−0.83*0.74) and 57% (1−0.95)/(1−0.95*0.96) of the total annual mortality in females and males, respectively. These figures seem large, but they are maximal values and clearly lower than the corresponding values of an American passerine with 85% mortality [2]. Thus, overall our data of Leislers' bat suggest that the migratory period was not a period with significantly high mortality. Similar studies in other bat species are necessary to gauge whether this is a general pattern in bat migration.

### Annual survival probability

Our study revealed that the estimated annual survival probabilities of the non-transient males were 0.91. This is a surprisingly high value that is similar to the survival probabilities of the greater horseshoe bat (*Rhinolophus ferrumequinum*) in Switzerland (0.91, [42]), which is supposed to be among the bat species with the highest longevity. The survival probability of males measured at the reproduction site in Germany was 0.69 [11], which is much lower than the estimate from the current study. If we had not considered transients, the annual survival would have been also 0.69 (CI: 0.54–0.83, estimated from a CJS model without transients), thus matching very well with the estimate from the reproduction site. Estimating the annual survival without taking into account transients is likely to reflect the mean annual survival of the overall population. However, our analyses provide evidence that there is strong heterogeneity among males either in survival or in site fidelity.

By contrast, the annual survival probability in non-transient females estimated in the current study was 0.61, thus less than female survival at the nursery colony (0.76) [11]. If transients were not taken into account, the annual survival would only be 0.54 (CI: 0.35–0.73). These results may be due to permanent emigration of females from our study population. This would imply that also non-transient females do not use the study site during their complete life, thus that they move to another site after having used the study site for some years. Overall our results suggest significant individual heterogeneity in either survival and/or, more likely, site fidelity and that heterogeneity is larger in males than in females. Survival probabilities of females from nursery colonies also differed between individuals that were born locally from those that were not, likely as a result of differential site use as discussed in Schorcht et al. [11]. It therefore appears that, similarly as at the reproduction sites, the use of sites outside the reproduction period differs individually, probably due to differential spatio-temporal habitat selection and migratory behaviour.

### Function of the study site in the annual cycle of Leisleri's bat

The seasonal variation in the number of captured individuals (fig. 1, panel A) showed presence of bats during autumn, winter and spring. In order to minimize disturbance during the delicate wintering period we scheduled only few controls in this season. With these occasional checks (Table S1 in File S1) it is difficult to be conclusive about whether the bats used the study site only as a stopover, or also as a wintering site. The mean number of captured bats that was present in the boxes was slightly lower in winter than during autumn (Fig. 1, panel A), suggesting that some individuals were not present during winter. Finally, during the winter control in year 2003 no female and only 9 males were present. Therefore, we think that the function of the study area is mixed, for some individuals (most likely males) it may well serve as a wintering site, while for others it is used as a stopover site only. Further studies during the winter period are needed for more conclusive results.

The capture-recapture data showed in addition that the marked individuals used the study site differentially: the majority of females and about half of the males used the site both during autumn and spring migration for several years. The annual survival probabilities of these philopatric individuals were either higher (males) or lower (females) than the annual survival estimates at the breeding area [11], suggesting that non-transient Leisler's bats showed high fidelity to the site. High fidelity to stopover or wintering sites is well known in migrating birds [43], [44], but has never been reported in bats. A critical resource needed by Leisler's bats is tree cavities. The availability of such cavities has low temporal variability, which might favour the evolution of site fidelity [45].

However, high site fidelity to the study site was not typical for all individuals. About half of the males were transients, that means they were present at the study site only shortly (i.e. at a single capture occasion) and never returned to it again. Transients at stopover site are common in migratory birds [46], [47]. We suggest three hypotheses to explain the high proportion of male transients. 1) Transients might be subordinate individuals that cannot defend a bat box. Presumably there are many younger individuals among them that are exploring potential mating roosts and are looking for occasional mating opportunities [48], [49]. Competition among males for optimal and possibly limiting resources is expected to be strong with the polygynous mating system of Leisler's bat [22] and may be the reason why there are more transients in males than in females. 2) The occurrence of transients could be the result of individual heterogeneity in the migration behaviour: some individuals may be migratory while others stay in the vicinity of the study site year round. Migratory individuals may then appear as transients at the study site. The fact that transients also appeared in spring is in favour of this explanation. 3) Individuals which appear as transients are not true transients in the sense that they are differentially philopatric to the study site, but have different survival probabilities. It is possible that these individuals have for whatever reason a lower survival probability, thus that there is strong individual heterogeneity in survival. These possible explanations are not mutually exclusive and further studies are necessary to shed more light on the reason for the occurrence of transients. We think that the first explanation is the most likely. By contrast, the occurrence of transients was less pronounced in females and it differed seasonally. Since females are not territorial, the first explanation of the male does not apply to females, but the other two explanations are possible.

Another function of the site was mating, inferred from the observation of harems in bat boxes. In autumn the mean proportion of males in boxes was lower than in the other seasons suggesting that Leisler's bats mated mainly in autumn, as is known for related bats species as well [23]. Temperate bats are known to spend the winter hibernating in mixed sex groups [22] and our data support this previous knowledge for Leisler's bats; similar proportions of males and females share roosts in winter.

## Conclusions

Our study revealed that certain patterns of migration ecology of Leisler's bat are similar to those of some birds: fidelity to a stopover or wintering site is well established and transients occur. In contrast to long-distance migratory birds, the migratory journeys are unlikely to bear significant costs in terms of survival in Leisler's bat. The stopover sites are used during autumn migration for mating in bats while mating in birds usually occurs in the breeding (most species) or wintering areas (e.g. *Anatidae*). The extended use of the non-breeding site during migration periods in spring and autumn and the site fidelity over years indicates a special importance of this and probably of other non-breeding sites in the life cycle of Leisler's bats. There is an urgent need to explore the spatial extent and the habitat requirements of non-breeding sites along the migration routes. Such non-breeding site should be considered in conservation action plans for migrating bat species. Our study provides evidence of strong individual difference in sites use in the course of the annual cycle. This poses challenges for modelling the dynamics of Leisler's bats and for efficient conservation, because populations are very difficult to define.

## Supporting Information

Figure S1 **Spring and autumn recapture probabilities.** Model averaged recapture probabilities of male (triangles) and female (dots) Leisler's bats. The vertical lines show the limits of the 95% credible intervals. Model averaged recapture probabilities of male (triangles) and female (dots) Leisler's bats (*Nyctalus leisleri*) in spring and autumn. The vertical lines show the limits of the 95% credible intervals.
(TIFF)

Table S1 Field work sessions within the study. Number of bat box controls performed during the study per year and per season. Winter refers to the period from November 16th to February 15th, spring to the period from February 16th to May 15th, summer to the period from May 16th to August 15th and autumn to the period from August 16th to November 15th). Note that winter in year *t* refers to mid November in year *t* to mid February in year *t*+1.
(DOC)

Table S2 Complete model selection results for survival and transience probabilities. Complete model selection results for survival (Survival model) and transience (Transience model) probabilities of Leisler's bat (*Nyctalus leisleri*) sampled in southern Switzerland, during the period 2001–2006 in relation to seasonality. Given are the model names, the model deviance (deviance), the model complexity (pD), the difference of the deviance information criterion between the current and the best model (ΔDIC) and the model weights (w_i_). The recapture model was in always the same, i.e. *p*(sex * year * season). For each survival and transient model the linear equation on the logit scale is given. μ_s_: mean survival for each sex; γ_s_: fixed seasonal effect on survival for each sex; σ^2^_s_: temporal variance of survival for each sex; η_s_: mean transience for each sex; λ_s_: fixed seasonal effect on transience for each sex; ς^2^_s_: temporal variance of transience for each sex; m: parameter refers to males only; f: parameter refers to females only.
(DOC)

File S1 **Combined supporting information file containing Tables S1 and S2.**
(DOC)

Text S1 **JAGS code of the most complex model.**: p(sex*year*season), μ_s_ + γ_s_ + ε_s,t_; ε_s,t_ ∼ N(0,σ^2^_s_), η_s_ + ω_s,t_; ω_s,t_ ∼ N(0,ς^2^_s_)
(DOC)
