# Supplementary material for: Seasonal Survival Probabilities Suggest Low Migration Mortality in Migrating Bats
Source: PLoS One. 2014 Jan 15;9(1):e85628. doi: 10.1371/journal.pone.0085628 (PMC3893227; doi:10.1371/journal.pone.0085628)
Supplement: Table S1 — Field work sessions within the study. Number of bat box controls performed during the study per year and per season. Winter refers to the period from November 16th to February 15th, spring to the period from February 16th to May 15th, summer to the period from May 16th to August 15th and autumn to the period from August 16th to November 15th). Note that winter in year t refers to mid November in year t to mid February in year t+1. (DOC) [file pone.0085628.s002.doc]

**Supporting Information**

Supporting Information, table S1

Field work sessions within the study.

| year | winter | spring | summer | autumn |
| --- | --- | --- | --- | --- |
| 2001 | 1 | 1 | 1 | 1 |
| 2002 | 1 | 1 | 1 | 1 |
| 2003 | 1 | 3 | 1 | 3 |
| 2004 | 0 | 3 | 1 | 2 |
| 2005 | 3 | 3 | 1 | 4 |
| 2006 | 3 | 6 | 6 | 6 |

Table S1. Number of bat box controls performed during the study per year and per season. Winter refers to the period from November 16th to February 15th, spring to the period from February 16th to May 15th, summer to the period from May 16th to August 15th and autumn to the period from August 16th to November 15th). Note that winter in year *t* refers to mid November in year *t* to mid February in year *t*+1.

Supporting Information, table S2

Complete model selection results for survival and transience probabilities.

| Survival model | Transience model | Deviance | pD | ΔDIC | w_i_ |
| --- | --- | --- | --- | --- | --- |
| μ_s_ + ε_s,t_; ε_s,t_ ~ N(0,σ^2^_s_) | η_s_ + λ_f_ + ω_s,t_; ω_s,t_ ~ N(0,V^2^) | 378.83 | 28.87 | 0.00 | 0.20 |
| μ_s_ + γ_f_ + ε_s,t_; ε_s,t_ ~ N(0,σ^2^_s_) | η_s_ + λ_f_ + ω_s,t_; ω_s,t_ ~ N(0,V^2^) | 378.98 | 29.45 | 0.73 | 0.14 |
| μ_s_ + γ_f_ + ε_s,t_; ε_s,t_ ~ N(0,σ^2^_s_) | η_s_ + ω_s,t_; ω_s,t_ ~ N(0,V^2^) | 379.00 | 30.28 | 1.58 | 0.09 |
| μ_s_ + ε_s,t_; ε_s,t_ ~ N(0,σ^2^_s_) | η_s_ + λ_f_ + ω_s,t_; ω_s,t_ ~ N(0,V^2^_s_) | 377.99 | 31.43 | 1.72 | 0.08 |
| μ_s_ + γ_f_ + ε_s,t_; ε_s,t_ ~ N(0,σ^2^_s_) | η_s_ + λ_f_ + ω_s,t_; ω_s,t_ ~ N(0,V^2^_s_) | 377.15 | 32.90 | 2.34 | 0.06 |
| μ_s_ + γ_f_ + ε_s,t_; ε_s,t_ ~ N(0,σ^2^_s_) | η_s_ + λ_m_ + ω_s,t_; ω_s,t_ ~ N(0,V^2^) | 379.57 | 30.55 | 2.42 | 0.06 |
| μ_s_ + ε_s,t_; ε_s,t_ ~ N(0,σ^2^_s_) | η_s_ + ω_s,t_; ω_s,t_ ~ N(0,V^2^) | 380.12 | 30.47 | 2.89 | 0.05 |
| μ_s_ + γ_s_ + ε_s,t_; ε_s,t_ ~ N(0,σ^2^_s_) | η_s_ + λ_f_ + ω_s,t_; ω_s,t_ ~ N(0,V^2^) | 379.99 | 31.29 | 3.58 | 0.03 |
| μ_s_ + γ_f_ + ε_s,t_; ε_s,t_ ~ N(0,σ^2^_s_) | η_s_ + λ_s_ + ω_s,t_; ω_s,t_ ~ N(0,V^2^_s_) | 377.71 | 33.72 | 3.73 | 0.03 |
| μ_s_ + ε_s,t_; ε_s,t_ ~ N(0,σ^2^_s_) | η_s_ + ω_s,t_; ω_s,t_ ~ N(0,V^2^_s_) | 379.00 | 32.57 | 3.87 | 0.03 |
| μ_s_ + ε_s,t_; ε_s,t_ ~ N(0,σ^2^_s_) | η_s_ + λ_m_ + ω_s,t_; ω_s,t_ ~ N(0,V^2^) | 380.94 | 31.17 | 4.42 | 0.02 |
| μ_s_ + γ_m_ + ε_s,t_; ε_s,t_ ~ N(0,σ^2^_s_) | η_s_ + ω_s,t_; ω_s,t_ ~ N(0,V^2^) | 381.43 | 30.72 | 4.45 | 0.02 |
| μ_s_ + γ_f_ + ε_s,t_; ε_s,t_ ~ N(0,σ^2^_s_) | η_s_ + ω_s,t_; ω_s,t_ ~ N(0,V^2^_s_) | 377.51 | 34.65 | 4.46 | 0.02 |
| μ_s_ + ε_s,t_; ε_s,t_ ~ N(0,σ^2^_s_) | η_s_ + λ_m_ + ω_s,t_; ω_s,t_ ~ N(0,V^2^_s_) | 379.27 | 33.52 | 5.08 | 0.02 |
| μ_s_ + γ_s_ + ε_s,t_; ε_s,t_ ~ N(0,σ^2^_s_) | η_s_ + ω_s,t_; ω_s,t_ ~ N(0,V^2^) | 380.30 | 32.55 | 5.15 | 0.02 |
| μ_s_ + ε_s,t_; ε_s,t_ ~ N(0,σ^2^_s_) | η_s_ + λ_s_ + ω_s,t_; ω_s,t_ ~ N(0,V^2^_s_) | 378.74 | 34.44 | 5.48 | 0.01 |
| μ_s_ + γ_s_ + ε_s,t_; ε_s,t_ ~ N(0,σ^2^_s_) | η_s_ + λ_s_ + ω_s,t_; ω_s,t_ ~ N(0,V^2^) | 380.84 | 32.37 | 5.51 | 0.01 |
| μ_s_ + ε_s,t_; ε_s,t_ ~ N(0,σ^2^_s_) | η_s_ + λ_s_ + ω_s,t_; ω_s,t_ ~ N(0,V^2^) | 379.70 | 33.51 | 5.51 | 0.01 |
| μ_s_ + γ_m_ + ε_s,t_; ε_s,t_ ~ N(0,σ^2^_s_) | η_s_ + λ_s_ + ω_s,t_; ω_s,t_ ~ N(0,V^2^) | 380.84 | 32.53 | 5.67 | 0.01 |
| μ_s_ + γ_f_ + ε_s,t_; ε_s,t_ ~ N(0,σ^2^_s_) | η_s_ + λ_s_ + ω_s,t_; ω_s,t_ ~ N(0,V^2^) | 379.59 | 33.85 | 5.74 | 0.01 |
| μ_s_ + γ_s_ + ε_s,t_; ε_s,t_ ~ N(0,σ^2^_s_) | η_s_ + ω_s,t_; ω_s,t_ ~ N(0,V^2^_s_) | 378.42 | 35.08 | 5.80 | 0.01 |
| μ_s_ + γ_s_ + ε_s,t_; ε_s,t_ ~ N(0,σ^2^_s_) | η_s_ + λ_f_ + ω_s,t_; ω_s,t_ ~ N(0,V^2^_s_) | 378.44 | 35.15 | 5.90 | 0.01 |
| μ_s_ + γ_s_ + ε_s,t_; ε_s,t_ ~ N(0,σ^2^_s_) | η_s_ + λ_m_ + ω_s,t_; ω_s,t_ ~ N(0,V^2^) | 380.88 | 32.93 | 6.11 | 0.01 |
| μ_s_ + γ_m_ + ε_s,t_; ε_s,t_ ~ N(0,σ^2^_s_) | η_s_ + λ_f_ + ω_s,t_; ω_s,t_ ~ N(0,V^2^_s_) | 379.56 | 34.30 | 6.16 | 0.01 |
| μ_s_ + γ_f_ + ε_s,t_; ε_s,t_ ~ N(0,σ^2^_s_) | η_s_ + λ_m_ + ω_s,t_; ω_s,t_ ~ N(0,V^2^_s_) | 377.98 | 35.92 | 6.20 | 0.01 |
| μ_s_ + γ_m_ + ε_s,t_; ε_s,t_ ~ N(0,σ^2^_s_) | η_s_ + λ_f_ + ω_s,t_; ω_s,t_ ~ N(0,V^2^) | 380.18 | 34.11 | 6.59 | 0.01 |
| μ_s_ + γ_m_ + ε_s,t_; ε_s,t_ ~ N(0,σ^2^_s_) | η_s_ + λ_m_ + ω_s,t_; ω_s,t_ ~ N(0,V^2^) | 382.12 | 32.57 | 6.99 | 0.01 |
| μ_s_ + γ_m_ + ε_s,t_; ε_s,t_ ~ N(0,σ^2^_s_) | η_s_ + λ_m_ + ω_s,t_; ω_s,t_ ~ N(0,V^2^_s_) | 380.92 | 34.28 | 7.50 | 0.00 |
| μ_s_ + γ_s_ + ε_s,t_; ε_s,t_ ~ N(0,σ^2^_s_) | η_s_ + λ_m_ + ω_s,t_; ω_s,t_ ~ N(0,V^2^_s_) | 379.20 | 36.01 | 7.50 | 0.00 |
| μ_s_ + γ_m_ + ε_s,t_; ε_s,t_ ~ N(0,σ^2^_s_) | η_s_ + ω_s,t_; ω_s,t_ ~ N(0,V^2^_s_) | 380.47 | 35.44 | 8.22 | 0.00 |
| μ_s_ + γ_s_ + ε_s,t_; ε_s,t_ ~ N(0,σ^2^_s_) | η_s_ + λ_s_ + ω_s,t_; ω_s,t_ ~ N(0,V^2^_s_) | 379.11 | 37.55 | 8.87 | 0.00 |
| μ_s_ + γ_m_ + ε_s,t_; ε_s,t_ ~ N(0,σ^2^_s_) | η_s_ + λ_s_ + ω_s,t_; ω_s,t_ ~ N(0,V^2^_s_) | 380.46 | 36.19 | 8.95 | 0.00 |

Table S2. Complete model selection results for survival (Survival model) and transience (Transience model) probabilities of Leisler’s bat (*Nyctalus leisleri*) sampled in southern Switzerland, during the period 2001-2006 in relation to seasonality. Given are the model names, the model deviance (deviance), the model complexity (pD), the difference of the deviance information criterion between the current and the best model (ΔDIC) and the model weights (w_i_). The recapture model was in always the same, i.e. *p*(sex * year * season). For each survival and transient model the linear equation on the logit scale is given. μ_s_: mean survival for each sex; γ_s_: fixed seasonal effect on survival for each sex; σ^2^_s_: temporal variance of survival for each sex; η_s_: mean transience for each sex; λ_s_: fixed seasonal effect on transience for each sex; V^2^_s_: temporal variance of transience for each sex; m: parameter refers to males only; f: parameter refers to females only.
