# Supplementary material for: Seasonal Survival Probabilities Suggest Low Migration Mortality in Migrating Bats
Source: PLoS One. 2014 Jan 15;9(1):e85628. doi: 10.1371/journal.pone.0085628 (PMC3893227; doi:10.1371/journal.pone.0085628)
Supplement: Text S1 — JAGS code of the most complex model.: p(sex*year*season), μs + γs + εs,t; εs,t ∼ N(0,σ2 s), ηs + ωs,t; ωs,t ∼ N(0,ς2 s) (DOC) [file pone.0085628.s005.doc]

Supporting Information, text S1

JAGS code of the most complex model: p(sex*year*season), μ_s_ + γ_s_ + ε_s,t_; ε_s,t_ ~ N(0,σ^2^_s_), η_s_ + ω_s,t_; ω_s,t_ ~ N(0,V^2^_s_)

model {

# PRIORS AND CONSTRAINTS

#------------------------------------------------ P ------------------------------------

for (t in 1:(n.occasions-1)){

for (j in 1:2){

p[j,t] ~ dunif(0, 1)

} #j

} #t

#---------------------------------------------- P H I ----------------------------------

for (t in 1:(n.occasions-1)){

phi[1,t] <- phi[2,t] * (1 - tau[1,t])

logit(phi[2,t]) <- lmean.phi[1] + eps[1,t]

eps[1,t] ~ dnorm(0, tau.eps[1])

phi[3,t] <- phi[4,t] * (1 - tau[2,t])

logit(phi[4,t]) <- lmean.phi[2] + eps[2,t]

eps[2,t] ~ dnorm(0, tau.eps[2])

} #t

for (t in 1:(n.occasions-1)){ # Prior for the transience probability. Note that tau[1,1] and tau[2,1] are not identifiable

logit(tau[1,t]) <- lmean.tau[1] + eps.tau[1,t]

eps.tau[1,t] ~ dnorm(0, tau.eps.tau[1])

logit(tau[2,t]) <- lmean.tau[2] + eps.tau[2,t]

eps.tau[2,t] ~ dnorm(0, tau.eps.tau[2])

}

for (i in 1:2){

lmean.phi[i] <- log(mean.phi[i] / (1 - mean.phi[i]))

mean.phi[i] ~ dunif(0, 1)

lmean.tau[i] <- log(mean.tau[i] / (1 - mean.tau[i]))

mean.tau[i] ~ dunif(0, 1)

tau.eps[i] <- pow(sigma.phi[i], -2)

sigma.phi[i] ~ dunif(0, 10)

tau.eps.tau[i] <- pow(sigma.tau[i], -2)

sigma.tau[i] ~ dunif(0, 10)

}

#-----------------------------------------------------------------------------------------

# LIKELIHOOD

# Define the multinomial likelihood

for (t in 1:(n.occasions-1)){

marr.mt[t,1:n.occasions] ~ dmulti(pr.mt[t,], r.mt[t])

}

for (t in 1:(n.occasions-2)){ # Because no relesases at second last occasion

marr.ft[t,1:n.occasions] ~ dmulti(pr.ft[t,], r.ft[t])

}

for (t in 2:(n.occasions-1)){

marr.m[t,1:n.occasions] ~ dmulti(pr.m[t,], r.m[t])

marr.f[t,1:n.occasions] ~ dmulti(pr.f[t,], r.f[t])

}

# Define the cell probabilities of the m-arrays

# Main diagonal

for (t in 1:(n.occasions-1)){

q.m[t] <- 1-p[1,t] # Probability of non-recapture for males

q.f[t] <- 1-p[2,t] # Probability of non-recapture for females

pr.m[t,t] <- phi[1,t]*p[1,t]

pr.mt[t,t] <- phi[2,t]*p[1,t]

pr.f[t,t] <- phi[3,t]*p[2,t]

pr.ft[t,t] <- phi[4,t]*p[2,t]

# Above main diagonal

for (j in (t+1):(n.occasions-1)){

pr.m[t,j] <- prod(phi[2,t:j])*prod(q.m[t:(j-1)])*p[1,j]

pr.mt[t,j] <- phi[1,t]*prod(phi[2,(t+1):j])*prod(q.m[t:(j-1)])*p[1,j]

pr.f[t,j] <- prod(phi[4,t:j])*prod(q.f[t:(j-1)])*p[2,j]

pr.ft[t,j] <- phi[3,t]*prod(phi[4,(t+1):j])*prod(q.f[t:(j-1)])*p[2,j]

} #j

# Below main diagonal

for (j in 1:(t-1)){

pr.m[t,j] <- 0

pr.mt[t,j] <- 0

pr.f[t,j] <- 0

pr.ft[t,j] <- 0

} #j

} #t

# Last column: probability of non-recapture

for (t in 1:(n.occasions-1)){

pr.m[t,n.occasions] <- 1-sum(pr.m[t,1:(n.occasions-1)])

pr.mt[t,n.occasions] <- 1-sum(pr.mt[t,1:(n.occasions-1)])

pr.f[t,n.occasions] <- 1-sum(pr.f[t,1:(n.occasions-1)])

pr.ft[t,n.occasions] <- 1-sum(pr.ft[t,1:(n.occasions-1)])

} #t

# Annual survival

ann.male <- mean.phi[1] * mean.phi[1]

ann.female <- mean.phi[2] * mean.phi[2]

}
